# LncRNA HOTTIP-Mediated HOXA11 Expression Promotes Cell Growth, Migration and Inhibits Cell Apoptosis in Breast Cancer

**DOI:** 10.3390/ijms19020472

**Published:** 2018-02-06

**Authors:** Yanqin Sun, Chao Zeng, Siyuan Gan, Hongmei Li, Ying Cheng, Dongjie Chen, Rujia Li, Wei Zhu

**Affiliations:** 1 Department of Pathology, Guangdong Medical University, Dongguan 523808, China; sunyanqin@gdmu.edu.cn (Y.S.); zengchao@gdmu.edu.cn (C.Z.); gansiyuan@gdmu.edu.cn (S.G.); lihongmei@gdmu.edu.cn (H.L.); chengying7554@163.com (Y.C.); lirujia@gdmu.edu.cn (R.L.); 2 Department of Radiotherapy, State Key Laboratory of Oncology in South China, Cancer Center, Sun Yat-Sen University, Guangzhou 510060, China; chendongj@sysucc.org.cn

**Keywords:** HOTTIP, breast cancer, growth, migration, apoptosis

## Abstract

As the most common cause of cancer death in women, the pathogenesis of breast cancer still remains unclear. Here, we reported a long non-coding RNA (lncRNA), HOTTIP (HOXA transcript at the distal tip), that may play an important role in the pathogenesis of breast cancer. Using gain-and-loss-of experiments in vitro and in vivo, we observed the marked upregulation of HOTTIP/HOXA11 in the breast cancer cell line, MCF-7, and the downregulation of HOTTIP or HOXA11, which might inhibit cell proliferation and migration but promote cell apoptosis in breast cancer MCF-7 cells. In addition, by further rescue experiments with HOXA11 overexpression, we uncovered a novel potential regulatory mechanism between HOTTIP and one of its physical HOXA clusters, HOXA11. Hence, HOTTIP may mediate, at least partly, HOXA11 expression involved in cell growth, migration, and apoptosis of breast cancer MCF-7 cells.

## 1. Introduction

Breast cancer is the most common causes of cancer death from gynecologic tumors, and is mostly treated with combined treatment modalities, including surgery, chemotherapy, radiotherapy, hormonotherapy, and molecular targeting treatments [1]. Due to difficulties in early detection, most breast cancers are diagnosed at an advanced stage and treated with palliative care, which may greatly reduce the quality of the patients’ lives [2]. Although a large number of studies have been carried out in the field of breast cancer biology recently, the pathogenesis of breast cancer still remains unclear. 

Non-coding RNAs account for more than 90% of the transcriptome without protein-coding potential [3]. Among them, long non-coding RNAs (lncRNAs) with lengths over 200 nucleotides have been identified to play crucial regulatory roles in tissue differentiation, proliferation, migration, invasion, and apoptosis [4]. Recent studies have indicated that lncRNAs can regulate the expression of some key oncogenes or tumor suppressor genes and affect the occurrence and development of tumors [5]. However, the prevalence and functional significance of lncRNA-mediated tumorigenesis in breast cancer still needs further investigation.

The lncRNA, HOTTIP (HOXA transcript at the distal tip), a newly identified lncRNA, is located at the 5′ end of the HOXA cluster [6]. It is reported as a key locus control element of HOXA genes by being brought into close proximity to them by chromosomal looping, and a number of studies have demonstrated that HOTTIP may play important roles in the differentiation, proliferation, and genome maintenance of various types of human cancers by regulating the expression of its neighboring HOXA genes [7]. For instance, Quagliata et al. proposed a functional role for HOTTIP in disease progression and suggested that it predict the outcome in hepatocellular carcinoma by regulating HOXA13 [8]. Although HOTTIP has been reported to be one of the predictors of breast cancer prognosis, the underlying role of HOTTIP in the pathogenesis of breast cancer still remains unknown [9]. Here, we report lncRNA HOTTIP, which is specifically amplified in a breast cancer cell line and is associated with breast cancer cell growth, cell cycle arrest, apoptosis, and migration, probably by partly mediating HOXA11 expression. 

## 2. Results

### 2.1. Determination of HOTTIP/HOXA11 Levels in Breast Cancer MCF-7 Cells

**Figure 1:**
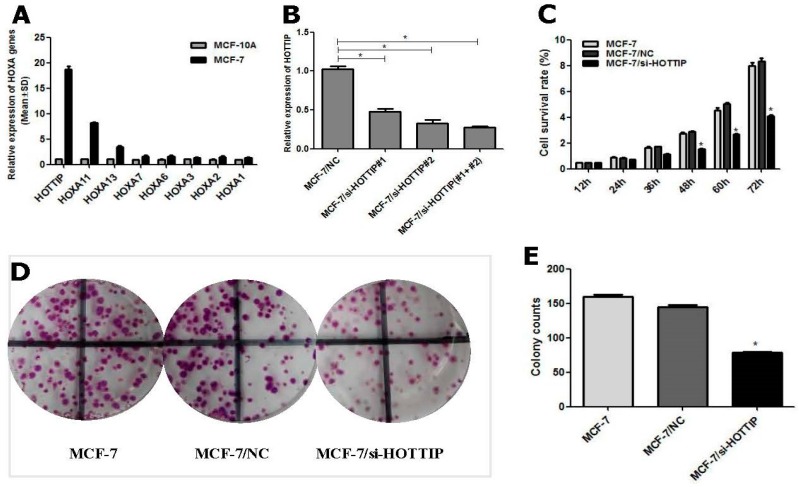

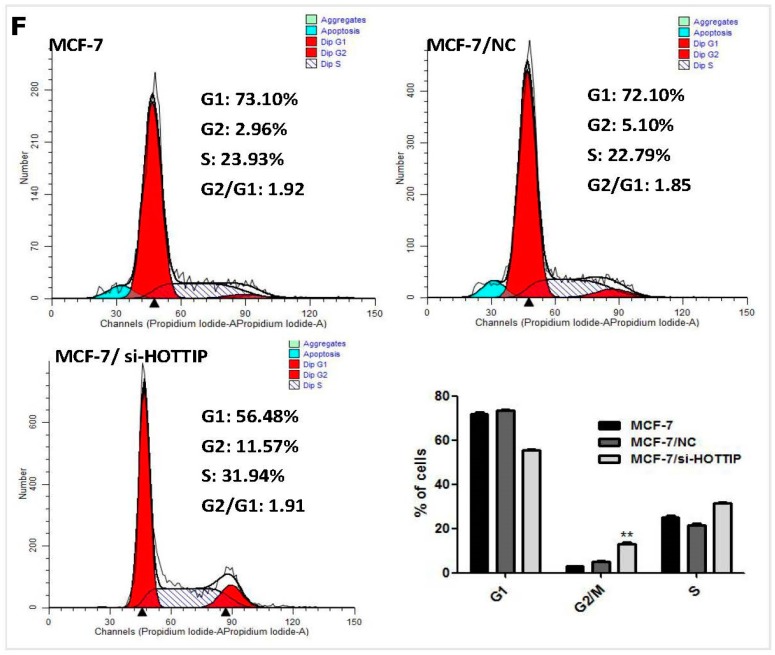
HOTTIP (HOXA transcript at the distal tip) may be involved in the proliferation, colony formation, and cell cycle arrest of breast cancer cells. (**A**): Expression of HOTTIP and HOXA gene clusters in MCF-7 and MCF-10A cells. (**B**): Effective RNAi sequences of HOTTIP knockdown were screened by RT-qPCR. (**C**): HOTTIP knockdown may inhibit cell proliferation of MCF-7 cells. (**D**): HOTTIP knockdown may inhibit colony formation ability of MCF-7 cells. (**E**): Quantitative results of cells colony formation with HOTTIP knockdown. (**F**): HOTTIP knockdown may lead to an increase in G2/M phrase cell amounts. NC (negative control) group stands for empty vector transfected control cells. * *p* < 0.05; ** *p* < 0.01.

Based on the literature reports and recent studies of our team, HOX genes have been found to be involved in the development and resistance of chemotherapeutic agents as a key oncogene [10,11,12,13]. Therefore, we used RT-qPCR technology to detect the expression of HOTTIP and its physical proximity to HOX genes in the breast cancer cell line, MCF-7, and the normal human mammary epithelial cell line, MCF-10A. The results showed that HOTTIP and HOXA11 were significantly upregulated in MCF-7 cells by more than 5-fold over the control group (Figure 1A).

### 2.2. Screening of HOTTIP/HOXA11 Interference Sequences

To manipulate HOTTIP levels in breast cancer cells, HOTTIP RNAi sequences (GenePharma, Suzhou, China) were transfected into MCF-7 cells. RT-qPCR analysis of HOTTIP levels was performed at 24 h after transfection and revealed that HOTTIP expression was effectively inhibited. The observed inhibition levels of HOTTIP expression were 52.0% by si-HOTTIP-1, 67.3% by si-HOTTIP-2, and 71.5% by si-HOTTIP-1 and si-HOTTIP-2 (Figure 1B). The combination of si-HOTTIP-1 and si-HOTTIP-2 was then subsequently used in the following loss-of-function studies. For stable HOTTIP RNAi effects, the RNAi sequences of the combination of si-HOTTIP-1 and si-HOTTIP-2 were packaged by a lentivirus vector for the following studies. 

### 2.3. HOTTIP Regulates Breast Cancer Cell Growth In Vitro and In Vivo

To investigate the effect of HOTTIP on the pathogenesis of breast cancer in vitro, Cell Counting Kit 8 (CCK-8) and plate colony formation assays were carried out in HOTTIP downregulated cells. CCK-8 assays revealed that HOTTIP knockdown reduced cell proliferation, compared with either of the control group (MCF-7 or MCF-7/NC) in MCF-7 cells (Figure 1C). The plate colony forming assay revealed that HOTTIP knockdown inhibited the colony formation ability of MCF-7 cells (Figure 1D,E), which is consistent with the result of the CCK-8 assay. To further investigate the growth inhibition observed following HOTTIP knockdown, cell-cycle profiles of HOTTIP knockdown cells were carried out by flow cytometry. The suppression of HOTTIP led to cell blockade characterized by phase G2/M block and an increase in the number of MCF-7 cells in the G2/M-phase (Figure 1F). 

**Figure 2:**
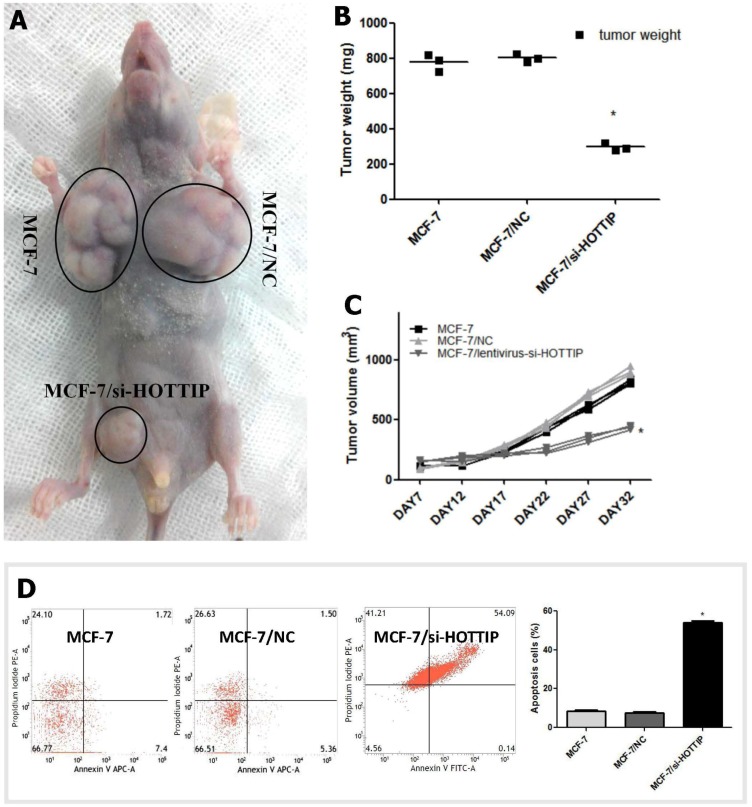

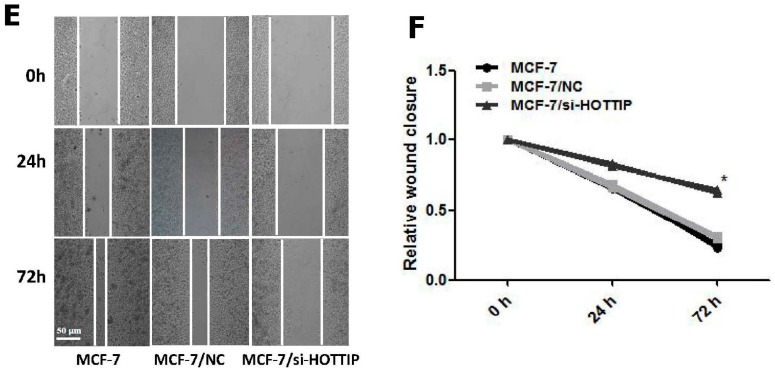
HOTTIP may promote cell growth in vivo, suppress cell apoptosis and promote cell migration in vitro in breast cancer cells. (**A**): Image showing excised tumors from tumor-bearing nude mouse for each treatment. (**B**): Volume change curve of each group measured on the indicated days. (**C**): Tumor weights of each group were determined. (**D**): HOTTIP knockdown may induce apoptosis of MCF-7 cells. (**E**): HOTTIP knockdown may inhibit cell migration ability of MCF-7. (**F**): Quantitative results of wound closure rate with HOTTIP knockdown in MCF-7 cells. * *p* < 0.05. Scale bar represents 50 μm.

The effect of HOTTIP in direct relation to breast cancer biology was further examined using an in vivo xenograft model in nude mice. As shown in Figure 2, tumor growth was most significantly inhibited in mice following HOTTIP knockdown treatment in MCF-7 cells compared with any other group (Figure 2A). After subcutaneous injection for 17 days, the mean tumor volume for the HOTTIP knockdown group was markedly smaller than any other group (Figure 2B). As expected, the tumor weight statistic of excised tumors showed a similar trend to that of tumor volume (Figure 2C). 

### 2.4. HOTTIP Suppresses Cell Apoptosis and Promotes Cell Migration In Vitro

Cell apoptosis assay by flow cytometry was carried out to determine the effect of HOTTIP on cell viability. The results showed that the fraction of late apoptotic cells in HOTTIP knockdown cells was significantly higher than the NC group (Figure 2D). Additionally, it should be noted that HOTTIP knockdown causes a considerable increase in the level of necrotic cells (Figure 2D).

As observed in Figure 2E, a scratch wound healing test was used to determine the effect of HOTTIP on cell migration. The results showed that HOTTIP knockdown led to a significant reduction of the wound closure rate in MCF-7 cells (Figure 2E,F). 

### 2.5. A Potential Bidirectional Regulation between HOTTIP/HOXA11 in MCF-7 Cells

**Figure 3:**
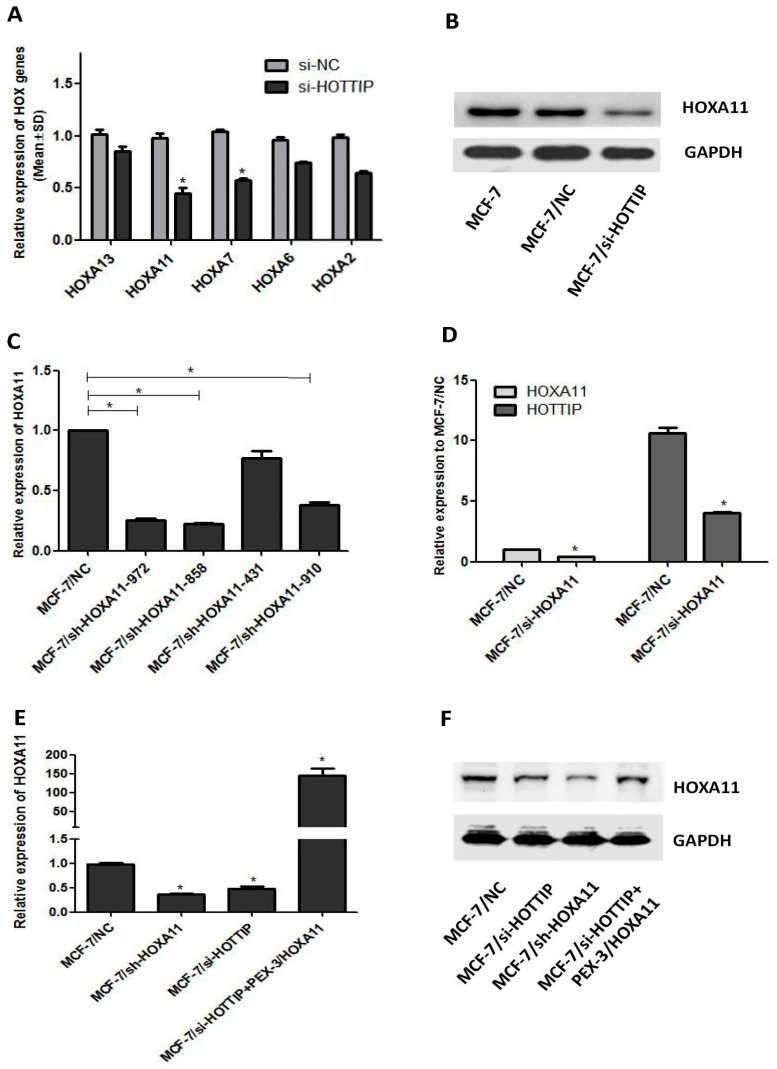
A potential bidirectional regulation loop between HOTTIP/HOXA11 in MCF-7 cells. (**A**): Expression of HOXA genes under HOTTIP knockdown in MCF-7 cells. (**B**): The effect of HOTTIP knockdown on HOXA11 expression at the protein level. (**C**): Screening of HOXA11 shRNA sequences by RT-qPCR. (**D**): HOXA11 knockdown decreased the expression of HOTTIP by RT-qPCR. (**E**): Rescue experiment of HOXA11 downregulation by HOTTIP knockdown by RT-qPCR. (**F**): Rescue experiment of HOXA11 downregulation by HOTTIP knockdown by Western blot. * *p* < 0.05.

The siRNA-mediated knockdown of HOTTIP resulted in a clear reduction of several HOX genes, particularly HOXA11 expression in Panc1 pancreatic cancer cells [14]. To explore whether the same holds true in a breast cancer cell line, we silenced HOTTIP expression through siRNAs in MCF-7 cells. Twenty-four hours post-siRNA delivery, HOTTIP downregulation resulted in 15% reduction of HOXA13, 55.34% reduction of HOXA11, 42.34% reduction of HOXA7, 15.67% reduction of HOXA6, and 35.67% reduction of HOXA2 levels (Figure 3A). Hence, HOTTIP may play a role in the regulation of several other HOX genes, especially HOXA11. We then focused on investigating the regulation between HOTTIP and HOXA11. Forty-eight hours post-siRNA delivery, at the protein level, we also observed HOXA11 reduction by HOTTIP knockdown (Figure 3B).

To gain further insight into the regulation of the HOTTIP/HOXA11 gene axis in breast cancer, we knocked down HOXA11 using shRNAs (short hairpin RNA, used for stable gene silencing) (Figure 3C) and chose shRNA #858 as the effective sequence of HOXA11 for the next experiments. In MCF-7 cells 24 h post-siRNA delivery, we observed a 48.67% reduction of HOXA11 levels (Figure 3D; shRNA #858). Interestingly, HOTTIP expression was also reduced by 62.27% (Figure 3D).

HOXA11 knockdown resulted in up to a 63.7% reduction of HOXA11 expression levels and concomitant reduction by 51.3% of HOTTIP (Figure 3E). In a parallel approach, we addressed the impact of HOXA11 overexpression on HOTTIP levels in the MCF-7 cell line. Using the PEX-3 vector for robust HOXA11 overexpression, we confirmed a rescue increase of HOXA11 caused by HOTTIP knockdown, both at the mRNA and protein levels (Figure 3E,F).

### 2.6. HOXA11 Partly Mediates the Effect of HOTTIP in Breast Cancer Pathogenesis

**Figure 4:**
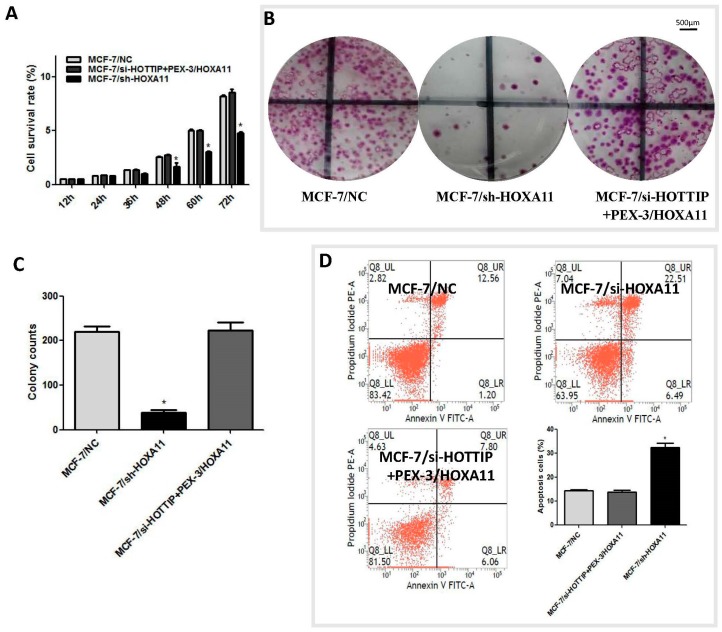

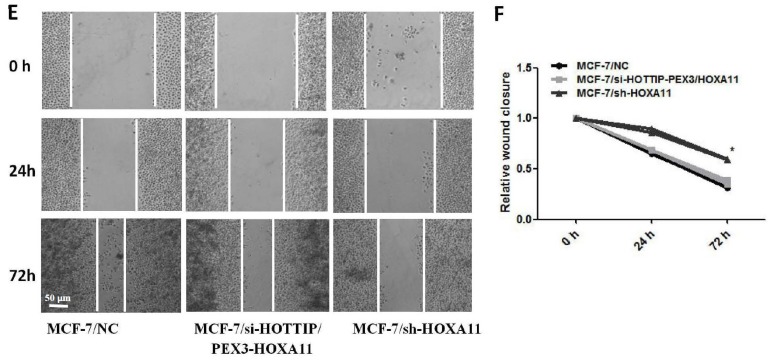
HOXA11 mediates the effect of HOTTIP on breast cancer pathogenesis. (**A**): Cell Counting Kit 8 (CCK-8) assay showed that HOXA11 overexpression could rescue the effect of HOTTIP/HOXA11 knockdown on MCF-7 cell proliferation. (**B**): Plate colony formation assay showed HOXA11 overexpression could rescue the effect of HOTTIP/HOXA11 knockdown on MCF-7 cell colony formation. (**C**): Quantitative results of MCF-7 cell colony formation in Figure 4B. (**D**): Flow cytometry showed HOXA11 overexpression could rescue the effect of HOTTIP/HOXA11 knockdown on MCF-7 cell apoptosis. (**E**): In vitro wound healing experiment showed HOXA11 overexpression could rescue the effect of HOTTIP/HOXA11 knockdown on MCF-7 cell migration; the figures of the wound healing experiment at 0 h, 24 h, and 72 h in MCF-7 cells are shown. (**F**): Quantitative results of the wound healing experiment in Figure 4E. * *p* < 0.05. Scale bar represents 50 μm.

Emerging evidence suggests that certain members of the HOXA cluster are involved in cancer progression. We hypothesized that HOTTIP might regulate the biological behavior of breast cancer via regulating HOXA11. To confirm this hypothesis, we evaluated the effect of HOXA11 overexpression on the knockdown of HOTTIP, as well as HOXA11 knockdown in MCF-7 cell pathogenesis. Interestingly, we found that as for HOTTIP knockdown, HOXA11 knockdown caused a significant reduction of proliferation (Figure 4A), colony formation (Figure 4B,C), and migration (Figure 4E,F) and promoted cell apoptosis (Figure 4D) in MCF-7 cells after shRNA delivery. Moreover, further rescue experiments revealed that HOXA11 overexpression could rescue the impact of HOTTIP knockdown in cell pathogenesis in breast cancer, and cell proliferation, colony formation, migration, and apoptosis rate were included (Figure 4A–F). Altogether, these results further substantiate the presumptive role of HOTTIP and HOXA11 in breast cancer cell progression and add new information supporting their interdependently regulated expression.

## 3. Discussion

Recent studies have shown that the dysregulated expression of lncRNAs in solid cancers reflects disease progression and may independently predict disease outcome [15,16]. HOTTIP, which lies at the 5’ tip of the HOXA locus and drives gene transcription by binding to the WD repeat domain 5 (WDR5)/mixed lineage leukemia (MLL) complex, has been identified as a critical factor with tumor progression in different cancers [7,8,14,17,18,19]. By way of interacting with the WDR5/MLL complex, HOTTIP is able to directly coordinate and control the activation of several of the 50 HOXA genes, HOXA11 included [14,19,20]. In the present study, we elucidated the involvement of lncRNA HOTTIP in breast cancer pathogenesis and further uncovered a potential bidirectional regulatory loop between HOTTIP and HOXA11 in this process.

This present study provides the first insight into the effect of HOTTIP in breast cancer cell behavior. Our results demonstrate a pivotal role of HOTTIP in breast cancer pathogenesis by applying loss-of-function experiments in vitro and in vivo. Firstly, by detecting the expression of HOXA genes, in both breast cancer MCF-7 cell line and normal mammary epithelial MCF-10A cell line, we found that both HOTTIP and HOXA11 were upregulated in MCF-7 cells in comparison with MCF-10A cells. Therefore, our data suggest that HOTTIP may be a novel potential oncogene in breast cancer. Secondly, to explore the role of HOTTIP in breast cancer biology, in vitro and in vivo experiments were carried out and it was found that HOTTIP knockdown inhibited breast cancer cell proliferation and migration, as well as resulted in cell blockade in the G2/M phase. Hence, the above findings suggest that HOTTIP may play a direct role in the modulation of multiple oncogenic properties and breast cancer progression. Thirdly, HOTTIP was revealed to be involved in breast cancer biology, at least partly, by mediating HOXA11 expression. However, HOTTIP downregulation may impair the expression of endogenous HOXA11 protein, while transfection of HOXA11 plasmids increased the expression of exogenous HOXA11 protein, so rescue experiments by HOXA11 overexpression at least caused the increase in HOXA11 expression, not only because the HOXA11 plasmid encodes for its coding sequence. Consequently, using a series of in vitro and in vivo experiments, our study suggested that HOTTIP is involved in breast cancer tumorigenesis through positively regulating HOXA11. 

As mentioned above, pioneering work in pancreatic cancer demonstrated the HOTTIP-targeted regulation of HOXA11 expression [14]. In accordance, we observed that HOTTIP knockdown decreased the expression of several HOXA genes, particularly HOXA11 in a breast cancer-derived cell line, but in contrast to liver cancer cells and consistent with pancreatic cancer cells, HOTTIP does not regulate the expression of HOXA13 [8]. Thus, the regulatory impact of HOTTIP on the HOXA locus is preserved also during breast cancer tumorigenesis. In addition, our work uncovered a previously unknown regulatory loop between HOTTIP and its target HOXA11. However, there are still some shortcomings in our work. First, the conclusion of this study will be more convincing once the clinical samples of patients with breast cancer are used to verify these results. Second, it would have been better to adopt more breast cancer cell lines in the study. Moreover, the mechanism of HOTTIP involved in breast cancer pathogenesis was not deep enough. 

In conclusion, using a combination of in vitro and in vivo approaches, our study highlights the molecular axes comprising HOTTIP and HOXA11 as key players in breast cancer progression. Future work will validate HOTTIP as a predictive bio-marker for breast cancer chemo-resistance, invasion, and metastasis. An in-depth study may provide novel insights into the mechanisms of breast cancer pathogenesis and possibly lead to the development of new therapeutic agents. 

## 4. Materials and Methods

### 4.1. Cell Culture

The human breast cancer MCF-7 and MCF-10A cell lines were maintained at 37 °C with 5% CO_2_ in a humidified atmosphere and grown in Dulbecco’s modified Eagle’s medium (HyClone, Los Angeles, CA, USA) supplemented with 10% fetal bovine serum (Gibco, CarIsbad, CA, USA) and were a gift from the Central Laboratory of Zhujiang Hospital, Southern Medical University (Guangzhou, China). 

### 4.2. Transient and Stable Transfection

The siRNA/shRNA vectors, pEX-3 HOXA11 overexpression vector and their respective NC (negative control) vectors were purchased from GenePharma, and transient and stable transfection was operated according to the respective vector’s instructions. The most effective interference sequences of HOTTIP/HOXA11, the combination of si-HOTTIP-1 and si-HOTTIP-2, as well as sh-HOXA11-858, were all selected by RT-PCR and then used for subsequent in vitro experiments. For stable transfection, the combination of si-HOTTIP-1 and si-HOTTIP-2 sequences was packaged by a lentivirus vector. The cloning site of pEX-3 HOXA11 overexpression vector lies at NheI/BamHI with a gene length of 942 bp (Host bacteria: Top10; plasmid: L4Y616-2). The related siRNA/shRNA sequences are listed in Supplemental Information.

### 4.3. Reverse Transcription Quantitative PCR (RT-qPCR)

RT-qPCR was used to detect the expression level of HOTTIP and other genes in breast cancer cells, according to the manufacturer’s instructions (TAKARA, Dalian, China). GAPDH was used as the control. The related primers are listed in Supplemental Information.

### 4.4. Western Blot Analysis

Western blot analysis was performed according to standard protocols, as described previously [21]. The antibody information is listed in Supplemental Information.

### 4.5. Flow Cytometric Analysis for Cell Cycle and Apoptosis

Cell cycle assays were performed after cells were fixed in 70% ethanol overnight at 4 °C and then were stained with propidium iodide. For apoptosis assays, cells were transfected with the combination of si-HOTTIP-1 and si-HOTTIP-2, and then all cells groups were treated with Adriamycin (conventional chemotherapy drug for breast cancer) (4 μM) for 24 h before being collected. An annexin V/propidium iodide detection kit (KeyGen, Nanjing, China) was used for the cell apoptosis assay. There was spontaneous green fluorescence of cells after transfection, so the gate detection by flow cytometry was regulated in cell apoptosis with negative staining and blank controls. The detailed procedure was carried out according to standard protocols described previously [22].

### 4.6. In Vitro Proliferation Assay

The cell counting experiment for the in vitro proliferation assay was performed using the Cell Counting Kit 8 (CCK-8, Dojindo, Kumamoto, Japan), according to the manufacturer’s instructions as described previously [23]. Plate colony formation experiments were also carried out according to standard protocols described previously [24].

### 4.7. In Vitro Wound Healing Experiment

MCF-7 cells were grown to confluence on 24-well plates. Consistently shaped wounds were made using a sterile 10 microliter pipette tip across each well. At least four images of the scraped area were captured using phase contrast microscopy after the scratch and at 0, 24, and 72 h. The whole procedure with three independent experiments was carried out based on the technique described previously [25]. The same scratched area was selected for the measurements at each time of study.

### 4.8. In Vivo Xenograft Model in Nude Mice

The xenograft tumor formation experiment was also carried out according to our standard protocols described previously [23]. Animals were randomly divided into the following groups (*n* = 3 mice per group): (a) MCF-7; (b) MCF-7/NC; and (c) MCF-7/si-HOTTIP. Once the average longest diameter of any group reached approximately 1 cm, mice were sacrificed, and the excised tumors were measured (Experimental animal ethics committee of Sun Yat-sen University, SYXK(YUE) 2012-0081, 28 July 2015).

### 4.9. Statistical Analysis

All experiments were run in triplicate. Data are represented as the mean ± standard deviation (mean ± SD). All statistical analyses were carried out using GraphPad Prism 5 Software (La Jolla, CA, USA). Statistical significance was analyzed by Student’s *t*-test or one-way ANOVA. *p* < 0.05 (compared with the negative control group) was considered significant and is marked with an asterisk in the figures.

## 5. Conclusions

By in vitro and in vivo experiments, our study indicated the role of lncRNA HOTTIP in breast cancer pathogenesis and its potential mechanism, by partly mediating its adjacent gene HOXA11. Further pathological and mechanical studies of this gene and its pathways involved are warranted in the future.

## Supplementary Materials

Supplementary materials can be found at http://www.mdpi.com/1422-0067/19/2/472/s1.

## Abbreviations

lncRNAs	long non-coding RNAs
HOTTIP	HOXA transcript at the distal tip
RT-qPCR	Reverse transcription quantitative PCR
CCK-8	cell counting kit 8
WDR5	WD repeat domain 5
MLL	mixed lineage leukemia
