# Supplementary material for: LncRNA HOTTIP-Mediated HOXA11 Expression Promotes Cell Growth, Migration and Inhibits Cell Apoptosis in Breast Cancer
Source: Int J Mol Sci. 2018 Feb 6;19(2):472. doi: 10.3390/ijms19020472 (PMC5855694; doi:10.3390/ijms19020472)
Supplement: Supplementary file 1 [file ijms-19-00472-s001.pdf]

Supplementary

LncRNA HOTTIP-Mediated HOXA11 Expression Promotes Cell Growth, Migration and Inhibits Cell Apoptosis in Breast Cancer

Primers:

HOTTIP Foward Primer(5'-3'): CCTAAAGCCACGCTTCTTTG

HOTTIP Reverse Primer(5'-3'): TGCAGGCTGGAGATCCTACT

HOXA11 Foward Primer(5'-3'): TGCCAAGTTGTACTTACTACGTC

HOXA11 Reverse Primer(5'-3'): GTTGGAGGAGTAGGAGTATGTCA

HOXA13 Foward Primer(5'-3'): CTGCCCTATGGCTACTTCGG

HOXA13 Reverse Primer(5'-3'): CCGGCGGTATCCATGTACT

HOXA7 Foward Primer(5'-3'): GCATAAGGACGAAGGTCCGA

HOXA7 Reverse Primer(5'-3'): AGACGCTTTTCCGACTGTCC

HOXA6 Foward Primer(5'-3'): TCCCGGACAAGACGTACAC

HOXA6 Reverse Primer(5'-3'): CGCCACTGAGGTCCTTATCA

HOXA3 Foward Primer(5'-3'): ATGCAAAAAGCGACCTACTACG

HOXA3 Reverse Primer(5'-3'): TACGGCTGCTGATTGGCATTA

HOXA2 Foward Primer(5'-3'): CCCCTGTCGCTGATACATTTC

HOXA2 Reverse Primer(5'-3'): TGGTCTGCTCAAAAGGAGGAG

HOXA1 Foward Primer(5'-3'): TCCTGGAATACCCCATACTTAGC

HOXA1 Reverse Primer(5'-3'): GCACGACTGGAAAGTTGTAATCC

GAPDH Forward Primers(5'-3'): GGGCTGCTTTTAACTCTG

GAPDH Reverse Primers(5’-3’): TGGCAGGTTTTTCTAGACGG

siRNA sequences of HOTTIP：

si-h-HOTTIP-1:

(positive-sense, 5'-3'): GCUGCUUUAGAGCCACAUA dTdT

(negative-sense, 3'-5'): dTdT CGACGAAAUCUCGGUGUAU

si-h-HOTTIP-2:

(positive-sense, 5'-3'): CCAGCUGCGAAUUCUUAAU dTdT

(negative-sense, 3’-5’):dTdT GGUCGACGCUUAAGAAUUA

si-h-HOTTIP-3:

(positive-sense, 5'-3'):CCUUGAUAUGCACGCAUAU dTdT

(negative-sense, 3’-5’): dTdT GGAACUAUACGUGCGUAUA

Vector type: LV3（H1/GFP&Puro）sequence: 5’ GCUGCUUUAGAGCCACAUA dTdT 3’

shRNA sequences of HOXA11:

sh-HOXA11-858:

(sense, 5'-3'): GCGUCUACAUUAACAAAGATT

(anti-sense, 5'-3'): UCUUUGUUAAUGUAGACGCTT

sh-HOXA11-431:

(sense, 5'-3'): GCAGUCUCGUCCAAUUUCUTT

(anti-sense, 5'-3'): AGAAAUUGGACGAGACUGCTT

sh-HOXA11-910:

(sense, 5'-3'): CACUGAUCGUCAAGUCAAATT

(anti-sense, 5'-3'): UUUGACUUGACGAUCAGUGTT

sh-HOXA11-972:

(sense, 5'-3'): GAGACCGUUUACAGUACUATT

(anti-sense, 5'-3'): UAGUACUGUAAACGGUCUCTT

Antibodies information:

Rabbit anti-human HOXA11 polyclonal IgG, (Biosis, bs-6666R)
